# Effect of Regular Exercise Program on Depression in Hemodialysis Patients

**DOI:** 10.1155/2015/182030

**Published:** 2015-01-06

**Authors:** Jahangir Rezaei, Alireza Abdi, Mansour Rezaei, Jafar Heydarnezhadian, Rostam Jalali

**Affiliations:** ^1^School of Nursing and Midwifery, Kermanshah University of Medical Sciences, Kermanshah 67817-53385, Iran; ^2^Department of Biostatistics, Kermanshah University of Medical Sciences, Kermanshah 67817-53385, Iran; ^3^School of Medicine, Kermanshah University of Medical Sciences, Kermanshah 67817-53385, Iran

## Abstract

*Background and Aim*. Depression is the most common psychological disorder in hemodialysis patients which decreases their quality of life and increases the mortality. This study was conducted to assess the effect of regular exercise on depression in hemodialysis patients. *Methods*. In a randomized clinical trial, 51 hemodialysis patients were allocated in two groups. Beck Depression Inventory (BDI) scale was used to assessing depression rate in participants. Designed program was educated using poster and face-to-face methods for case group. Intervention was carried out three times a week for ten weeks. At the beginning and the end of the study, depression rate of the subjects was assessed. Data was analyzed by SPSS16 software and descriptive and inferential statistics. *Findings*. According to the results of this study, there were no differences between case and control groups in depression rate at the beginning of the study, but there was significant difference after intervention (*P* = 0.016). In the beginning of the study, the mean and SD of depression in case group were 23.8 ± 9.29 and reduced to 11.07 ± 12.64 at the end (*P* < 0.001). *Conclusion*. The regular exercise program could reduce the depression in hemodialysis patients; therefore it is suggested for training this program for hemodialysis patients. This trial is registered with Iranian Registry of Clinical Trial (IRCT) number IRCT201205159763N1.

## 1. Introduction

Depression is the most common psychological disorder in hemodialysis patients, the various studies reported its range 25–70 percent [1, 2]. Depression underwent all aspects of the life in hemodialysis patients; some of these negative effects are reducing appetite, deteriorating physical power and motivation, impaired in blood urea and potassium, defect in regulation of blood pressure, and failure of supportive treatments [3]. Depression in hemodialysis patients is associated with increasing suicidal thoughts and weight loss [4]. Depressed hemodialysis patients have more admission and hospitalization in emergency room than nondepressed patients [5]. Mortality rate in depressed hemodialysis patients is 6.5 times higher than nondepressed, and attempting for kidney transplantation is 4.5 times less [6].

The hemodialysis patients rarely refer to treat their depression and other psychological disorders, and health care workers in therapeutic systems have little attention to this issue [7]. Because of the high prevalence of complications related to using of antidepressant agents such as production of blood toxic metabolites, cardiovascular disorders, vomiting, nausea, hepatotoxicity, risk of bleeding, and drug interaction, treating the depression in hemodialysis patients is facing several constraints [8].

In recent years, many studies stressed on nonmedical treatments to cure depression in hemodialysis patients instead of drug therapy; some of these methods are psychological, behavioral, modified regimens, supportive efforts by families, hypnotism, and muscle relaxation and meditation [9, 10], regarding insufficient evidences and need for facilities and special tools; these approaches are not applied as routine care extensively [8].

Exercise and physical activity, as a nonpharmacological care, are suggested to treat or help to cure major depression [11–13], but there are few studies and paradoxical results related to the effect of exercise on depression in hemodialysis patients [14–16]. Considering depression complications in hemodialysis patients, special limitations to their treatment (such as economical and social problems) and lack of attention in healthcare workers to this issue, this study was aimed at determining the effect of regular exercise on depression in hemodialysis patients.

## 2. Method

The study design is clinical trial and population was all of chronic renal failure (CRF) patients under treatment of hemodialysis in Emam Reza Hospital, an educational center affiliated to Kermanshah University of Medical Sciences (KUMS) of Kermanshah in the west of Iran. The hemodialysis center in this study has 35 active beds and hemodialysis procedure is performed three times a day. All of the patients are 300 persons. The samples include 70 CRF patients that were recruited by convenience sampling and then by randomizing approach, they were divided into two groups as a case and control (every group has 35 patients). Estimating the number of samples according to statistical formula [17], *α* = 0.05, *β* = 0.1, and other similar studies [14, 16] were 23 individuals in each groups, but due to possibility of attrition it was increased to 35 people (totally 70 individuals). Inclusion criteria were age 15–65 yrs, undertreatment of hemodialysis for at least during 3 months, not having progressive cardiovascular or respiratory diseases, lake of restricting musculoskeletal disorder, having physical power to exercise activity, not using any medicine or other procedures to treating depression, and undertreatment of hemodialysis 2 or 3 times a week. Exclusion criteria also include not performing the exercise program for 3 times continuingly or 5 times alternatively, dissatisfaction for continuing collaboration, and problematic hemodynamic instability. The instrument was a two-part questionnaire, including demographic characteristics and Beck Depression Inventory (BDI) for evaluating patient's depression; characteristics data have covered information about age, gender, dialysis duration, level of education, marital status, and causes of renal failure. BDI questionnaire has been used for assessing the depression of hemodialysis patients and validated in the same studies in Iran by Taraz et al. in Tehran [18] and Roozbeh et al. in Shiraz [19]. In our study also for assessing internal reliability, Cronbach's alpha of BDI was measured and it was 0.9 for control group, 0.86 for case group, and 0.89 for all patients. BDI has 21 questions and 0 to 3 scores for each one, total score is 0 to 63. Sum of the questions scores represents the rate of depression in each patient, the higher score indicating the more depression. Ethical considerations were permission from the ethics committee of vice research of center of KUMS with approving number 7.420.1394 in May 8, 2012, and written informed consent from participants. Assurance about anonymity and confidentiality of information was given to patients. Data was gathered with referring to the hemodialysis Center and by completing the questionnaire. Responding to questions of BDI test was performed by patients who were literate and not having visual impairment, but in illiterate and patients with vision disorders, the questions were read by researcher and the answers were written identically. After dividing the samples into two groups case and control randomizing (each one 35 individuals) and data collection, the exercise program was educated for all patients of case group via face-to-face method and poster in setting of hemodialysis ward and waiting room by researchers; then once the patients did exercise movements step by step, if there were any difficulties in performing the exercise, the education was continued until patients mastering. After assurance related to patient's ability for doing exercise program, the posters which contain pictures, sequences, and descriptions of all movements were given to cases group, suggesting them for performing the exercises three times a weeks for ten weeks in the days without dialysis sessions.

The exercise planning contains four types of exercise movements with less than moderate intensity. This program was designed through consulting with specialists in nephrology and exercise physiology and also using authentic sport books. Safety of the exercise program for hemodialysis patients was approved by the mentioned specialists. The movements of program include joints warming actions, stretching exercises, motions of lower back muscles and abdomen, and deep breathing exercises. In given poster the time of each exercise and rest between them had been written; this program was the anaerobic exercises; therefore it did not interfere with hemodynamic of patients, and they could perform it easily. The exercise plan was done three times a week for ten weeks at home, and every session has taken about 35 minutes. To ensure proper conducting of exercises program by hemodialysis patients, checking was done by calling to patients two times a week and visiting them once in hemodialysis ward; if there was a problem with the program, necessary explanations were given and it would be solved. There was no intervention on control group for treating depression during the study other than the routine procedures of hemodialysis ward.

At the end of the study, again, the rate of patient's depression (case and control groups) was measured by BDI scale. Data was analyzed by SPSS16. Descriptive statistics were used for estimating mean, standard deviation, frequency, and percent of data and chi-square test for assessing the relationship between qualitative variables. Independent *t*-test to compare age and dialysis duration between two groups, paired *t*-test for comparing the mean of depression number before and after intervention, and two-way ANOVA test were applied for examining the depression rate between case and control groups at the beginning and the end of study, in the two-way ANOVA test sex variable, because of differences in two groups, was considered as confounding variable. For evaluating of normality of depression rate Shapiro-wilk test was used. Significance level of tests was 0.05.

## 3. Findings

Of 70 participant patients of the study, 10 people from case group (4 males and 6 females) and 9 from control group (3 females and 6 males) were omitted from the study during study period; excluding causes were boring (5 individuals) and muscle cramp (4 individuals) in case group and traveling (5 individuals) and lack of cooperation in completing of the questionnaire (3 individuals) and kidney transplantation (1 individual) for control group, respectively. One of the patients in case group has died during surgery operation. Of 51 patients 36 people were male; mean and standard deviation (SD) of age were 43.27 ± 12.94. Majority of participants (76.9%) were married and about 90% of them had diploma and lower. There was no significance difference between case and control groups in terms of the variables such as age, dialysis duration, hemodialysis time, education level, and marital status (*P* > 0.05), but sex difference was significant after excluding cases from the study (*P* = 0.018) (Table 1).

At the beginning of study mean and SD of depression rate in case and control groups were 23.80 ± 9.29 and 19.3 ± 12.98, respectively, and there was no difference between case and control depression by two-way ANOVA test (*F* = 0.945  *P* = 0.336), in this stage also the mean of depression score according to sex variable (*F* = 0.034  *P* = 0.843) and interaction between sex and study groups (*F* = 0.133  *P* = 0.777) was not different. Mean and SD of depression score of case group have reduced to 11.07 ± 12.64 that this decrease in comparison with preintervention period was significant by paired *t*-test (*P* > 0.001), but in control group depression rate has been raised to 26.11 ± 13.72 (*P* = 0.002). Based on two-way ANOVA test the mean and SD of depression between case and control group differ after intervention and excluding the effect of sex variable (*F* = 6.48  *P* = 0.016) (Table 2), and also, in this test, mean and SD of depression differ according to sex (*F* = 1.58  *P* = 0.215) and interaction between sex and study groups (*F* = 0.555  *P* = 0.460).

## 4. Discussion

Considering the results of this study, regular exercise program had an important role in decreasing of depression in hemodialysis patients. The findings of a research in Turkey (2004) have indicated that walking 3 times a week improves the physical and psychological states of hemodialysis patients [20]; in Millagerdy et al. Study that assessed the effect of regular exercise on depression in 8–12 yrs hemodialysis patients, effectiveness of exercise and physical activity on depression in these clients was demonstrated [16]. Other researches such as Milani et al. in USA [21] and Oeland et al. in Denmark [22] also indicate the efficacy of exercise on major depression in heart failure patients, which are consistent with the results of this study. But in the Suh et al. [14] and Arcos-Carmona et al. [15] studies, exercise and physical activity have no effect on depression in hemodialysis patients.

It seems that designing exercise program in terms of timing, intensity, sequence of movements, being aerobic or resistance, running time a week, and cultural and social backgrounds of samples such as viewpoints to disability, underlying disease conditions and economic status, has important role on effectiveness of exercise in depression of hemodialysis patients; therefore difference in results of various researches can be investigated in sociocultural factors, type of exercise program, and studies method. In Ribeiro et al. research although resistance exercise planning during the hemodialysis sessions improved physical and psychological conditions but by intensification of exercises, along with its effects, some complications such as protein degradation, hypercalcemia, anemia, bone malformation, high blood pressure, muscle cramps, and central nerve system stimulation have emerged [23], so using exercises devices such as bodybuilding tools, treadmill, and stationary bicycle, as well as the problems and costs for preparation of them, needs coach and instructor for educating patients in order to reduce the complications and malformations; also there are not enough time and space for applying the sport devises in most hemodialysis patients; therefore these patients are faced with many constraints. Cheema and Singh expressed that the lack of coherent and workable exercise program and failure in methodology of trials are the main cues of no performing of exercise in hemodialysis patients routinely [24]. The criteria for proper exercise in hemodialysis patients in viewpoint of Ribeiro et al. include patient's compliance, feeling comfort with program, attention to social and familial factors, and life style [23]. It is believed that the efficacy of exercise program in this study can be due to strengthening muscle power, improving joints flexibility, reducing musculoskeletal pain, enhancing appetite and nutrition quality, feeling improvement and independence, self-care ability, getting well sleep and rest, and reducing fatigue, for hemodialysis patients. These effects allow the patients carry out their daily activities more effectively and so they are less dependent. In viewpoints of Kiuchi et al. the effect of exercise on depression is the result of increasing blood supply and neurogenesis in brain hippocampus, which acts like antidepressant agents [25]; Koeh et al. has indicated beta-endorphin produced by exercise plays an important role in construction of hippocampal neurons [26]. Animal and in vitro studies also have approved effect of beta-endorphin on decreasing depression [27]. In this regard, Krogh et al. stated the changing of growth and cortisol hormones following the exercise and improving the depression symptom in patients [28]. With respect to the positive features of the exercise program in this study like low intensity, simplicity, no need for spending extra costs and any equipment, and independence in its performing, the planning was highly acceptable and feasible for hemodialysis patients, so it can be easily taught in hemodialysis wards.

According to the findings, the rate of depression in controls group has been raised at the end of study, and its causes are not known for researchers accurately. Although some studies have pointed out about variation of catecholamine, melatonin, and serotonin in different session of year and effect on human temper [29],regarding randomizing of groups and the same conditions for them during the study period, rising of depression in control group is not justified, it shows the factors such as repeated referral to hemodialysis, fatigue following treatments, economic problems (such as rising prices of medicines and equipment and rumors about them being rare), and family issues can be cause of this subject, so it is suggested for doing more qualitative and quantitative studies about experience or process of depression developing and relationship between depression with contextual factors in hemodialysis patients.

## 5. Conclusion

In this study regular exercise program has reduced depression in hemodialysis patients. This exercise program has been designed considering all limitations (such as lack of space and facilities, inability for going to the gym, transportation difficulties, poor financial resources to treating depression and referring to psychologist, and lack of coach and qualified trainer) for performing the exercise in hemodialysis patients. Therefore it is suggested for performing and training the regular exercises as routine care.

*Limitations*. There were many limitations in this study; first some of the factors such as kt/v, economical status, existence or no supporter in family, nutritional status, and anemia which affect on depression in hemodialysis patients were not measurable and to thwart of these factors randomization was done. Second the attrition rate was high and increasing the depression rate in control group was unknown; the reasons of patient excluding from the study have been mentioned, but regarding similarity of remaining patients in terms of characteristics information and thwart of confounding factor by using two-way ANOVA, it can be said that the results have acceptable authenticity. But also it is recommended for carrying out more accurately additional qualitative and quantitative studies related to effect of exercise on depression in hemodialysis patients.

## Figures and Tables

**Table 1:** Distribution of frequency and frequency percent of case and control groups according to the variables of marital status, sex, educational level, hemodialysis time, hemodialysis duration, and age.

Demographic characteristics	Group name		
	Case number (percent)	Control number (percent)	Statistical test
Marital status			
Married	20 (80)	20 (76.9)	*χ* ^2^ = 0.71; *P* = 0.789
Single	5 (20)	6 (23.1)	
Sex			
Male	21 (84)	14 (53.8)	*χ* ^2^ = 4.38; *P* = 0.018^*^
Female	4 (16)	12 (46.2)	
Educational level			
Elementary and junior	11 (44)	18 (69.3)	*χ* ^2^ = 4.95; *P* = 0.292
High school	11 (44)	5 (19.2)	
Academic	3 (12)	3 (11.5)	
Hemodialysis time			
Morning	16 (64)	17 (65.4)	*χ* ^2^ = 4.77; *P* = 0.092
Evening	6 (24)	9 (34.6)	
Night	3 (12)	0 (00.0)	

Age and dialysis duration	Mean ± SD	Mean ± SD	Statistical test
Age (year)	43.96 ± 7.86	42.61 ± 12.67	*t* = 1.34; *P* = 0.653
Hemodialysis duration (year)	3.56 ± 3.24	2.96 ± 2.25	*t* = 0.786; *P* = 0.446

^*^Is statically significant.

**Table 2:** Comparison of the mean depression score in the case and control groups at the baseline and end of the study.

Assessment time of depression	Groups		
	Case Mean ± SD	Control Mean ± SD	Statistical test
Baseline	23.80 ± 10.29	19.23 ± 12.98	*F* = 0.945; *P* = 0.336
End	12.64 ± 11.07	26.11 ± 13.72	*F* = 6.48; *P* = 0.016^*^
Statistical test	*t* = 6.25; *P* < 0.001^*^	*t* = 3.46; *P* = 0.002^*^	

^*^Is statically significant.
